# The abscopal effect of immune-radiation therapy in recurrent and metastatic cervical cancer: a narrative review

**DOI:** 10.3389/fimmu.2023.1201675

**Published:** 2023-07-19

**Authors:** Luc Ollivier, Camille Moreau Bachelard, Emmanuelle Renaud, Estelle Dhamelincourt, Francois Lucia

**Affiliations:** ^1^ Department of Radiation Oncology, Institut De Cancérologie De L’Ouest (ICO), Saint-Herblain, France; ^2^ Department of Medical Oncology, Institut De Cancérologie De L’Ouest, Saint-Herblain, France; ^3^ Department of Medical Oncology, CHRU Morvan, University Hospital, Brest, France; ^4^ Radiation Oncology Department, University Hospital, Brest, France; ^5^ LaTIM, INSERM, UMR 1101, Univ Brest, Brest, France

**Keywords:** abscopal effect, immunotherapy, radiation therapy, cervical cancer, immune checkpoint inhibitors, metastastic cancer

## Abstract

Despite human papillomavirus vaccination and screening, in about 5% of cases, cervical cancer (CC) is discovered at an initial metastatic stage. Moreover, nearly one-third of patients with locally advanced CC (LACC) will have a recurrence of their disease during follow-up. At the stage of recurrent or metastatic CC, there are very few treatment options. They are considered incurable with a very poor prognosis. For many years, the standard of care was the combination of platinum-based drug and paclitaxel with the possible addition of bevacizumab. The most recent years have seen the development of the use of immune checkpoint inhibitors (ICIs) (pembrolizumab, cemiplimab and others) in patients with CC. They have shown long term responses with improved overall survival of patients in 1st line (in addition to chemotherapy) or 2nd line (as monotherapy) treatment. Another emerging drug is tisotumab vedotin, an antibody-drug conjugate targeting tissue factor. Radiation therapy (RT) often has a limited palliative indication in metastatic cancers. However, it has been observed that RT can induce tumor shrinkage both in distant metastatic tumors beyond the radiation field and in primary irradiated tumors. This is a rarely observed phenomenon, called abscopal effect, which is thought to be related to the immune system and allows a tumor response throughout the body. It would be the activation of the immune system induced by the irradiation of cancer cells that would lead to a specific type of apoptosis, the immunogenic cell death. Today, there is a growing consensus that combining RT with ICIs may boost abscopal response or cure rates for various cancers. Here we will review the potential abscopal effect of immune-radiation therapy in metastatic cervical cancer.

## 1 Introduction

Although considerable progress has been made in the prevention and treatment of this challenging disease, large populations in more rural and underserved regions continue to have much lower rates of vaccination. Thus, According to GLOBOCAN 2020, cervical cancer is one of the most common cancers among women worldwide, after breast, colon, and lung cancer, respectively. It is most common in developing countries, accounting for more than 85% of diagnosed cases (1). In developing countries where routine screening is not available, more than 70% of CC are diagnosed in advanced or metastatic stages (2, 3). In developed countries, in about 5% of cases, cervical cancer (CC) is discovered at an initial metastatic stage. Moreover, the number of new cases of locally advanced cervical cancer (LACC), also known as International Federation of Gynecology and Obstetrics (FIGO) 2018 stage IB3 and IIA2-IVA, is stable due to lack of widespread vaccination and incomplete compliance with screening. Concurrent chemoradiation therapy (CCRT) is the standard of care for patients with LACC. Despite considerable progress and optimization of treatments over the past 20 years, nearly one-third of patients will experience a recurrence of their disease despite the initial optimal management (4). Most patients with recurrent or metastatic disease will be treated with systemic therapy including chemotherapy (CT) plus or minus an angiogenesis inhibitor. However, HPV DNA is detected in over 99% of cervical cancer biopsies with the majority being of types 16 and 18. The E6 and E7 oncogenes are primarily responsible for the transformation of HPV-infected cervical keratinocytes. HPV-associated tumors always express these viral antigens which are attractive targets for immunotherapy (IT) (5).

Currently, the role of radiation therapy (RT) in the management of CC is mainly limited to the locally advanced stage. Cisplatin-based chemoradiation therapy (CRT) followed by Image Guided Adaptative Brachytherapy (IGABT) is the gold standard for FIGO 2018 stage IB3–IVA, regardless of histological subtype (6). However, stereotactic body radiotherapy (SBRT), for curative treatment of oligometastatic patients or palliative treatment in polymetastatic disease, has developed quickly in recent years. Its potential immunosensitizing effect, including the abscopal effect, raises the question of its association with IT in metastatic stages (7, 8).

In this review, we discuss the current status of IT for patients with primary metastatic or recurrent CC and the relevance of combining RT for its immunomodulatory effect.

## 2 Systemic treatment for recurrent or metastatic cervical cancer

### 2.1 Standard of care before immunotherapy

Since the results of the phase II trial Gynecologic Oncology Group (GOG)-26 the standard of care for recurrent disseminated CC has been cisplatin-based CT (9). Cisplatin combined with either paclitaxel, gemcitabine, topotecan, or vinorelbine was studied in the GOG-204 trial. Although the cisplatin-paclitaxel combination tended to have better results, no statistically significant difference was found between the different doublets. Despite these negative results, this combination was considered as the standard of care, particularly in women who had not received prior cisplatin-based therapy (10). In a non-inferiority study, 253 patients with recurrent stage IVB CC were randomized to paclitaxel plus either carboplatin or cisplatin. The combination of paclitaxel and carboplatin was non-inferior to the doublet paclitaxel and cisplatin in terms of PFS and OS, and showed a significant reduction in toxicity. However, in the subgroup of patients who had not previously received cisplatin the cisplatin-based doublet was superior (11).

Bevacizumab, a humanized monoclonal antibody targeting VEGF, is the leading antiangiogenic therapy studied in patients managed for CC (12). A randomized phase III trial showed an improvement in both PFS [8.2 versus 5.9 months; hazard ratio (HR) 0.67, 95% confidence interval (CI) 0.54-0.82] and OS (17.0 versus 13.3 months; HR 0.71, 95% CI 0.54-0.95) with the combination of CT (paclitaxel and cisplatin or topotecan) and bevacizumab (13, 14). As such, these combinations have been approved by the Food and Drug Administration (FDA) and the European Medicines Agency (EMA) for the treatment of patients with metastatic or recurrent CC.

### 2.2 Immunotherapy in metastatic cervical cancer

In recent years, several studies have focused on the use of IT for the treatment of CC because of evidence of an immune system response related to interactions with HPV infection (15).

#### 2.2.1 Rationale

HPV induces an immunosuppressive tumor microenvironment (TME) and deficient immunosurveillance by upregulating TGF-β via regulatory T (Treg) cells and changing the cytokine profile from a T helper 1 (Th1) profile to a Th2 profile (16). Moreover, some immune system inhibitory molecules such as Programmed cell death-1 (PD-1)/Programmed cell death ligand1 (PD-L1), are expressed by CC cells whereas they are not in healthy cervical tissue. However, PD-L1 expression seems to be more frequent in SCC (19% to 88%) than in adenocarcinoma (14%) (17, 18). Other immunomodulatory molecules such as cytokines (TGF-β, IL-10), cytotoxic T-lymphocyte antigen 4 (CTLA-4) and surface receptors (TIM3) have also been identified (19, 20). It was also shown that CD8+, CD4+ and Treg cells were more frequently found in the TME in CC than in normal cervical tissue with a significant decrease in survival (21, 22). Finally, studies have shown an increase in the total mutational burden (TMB) rate in CC (approximately 5-6 mutations per megabase) (23, 24). Thus, immune checkpoints inhibitors (ICIs) including inhibitors of PD-1 and PD-L1, as well as inhibitors of CTLA-4 have been evaluated in several CC trials (5, 25).

#### 2.2.2 Monotherapy

Monotherapy showed a lack of significant single-agent activity.

#### 2.2.3 PD-1 inhibitors

Pembrolizumab, balstilimab and nivolumab were evaluated in phase II studies for recurrent or metastastic CC in the second line-treatment. They found objective response rates (ORRs) of 12%, 15%, 26% respectively (26–30).

Cemiplimab was evaluated in a phase III study where 608 patients with recurrent or metastatic CC after platinum-based CT were randomized between cemiplimab or CT of investigator’s choice (31). Cemiplimab showed a significant improvement in overall survival, 12.0 versus 8.5 months, HR= 0.69 (0.56-0.84), p<0.0001.

#### 2.2.4 Anti CTLA-4

Forty-two patients with a recurrent or metastatic CC have received ipilimumab in a phase I-II study. The mPFS and mOS were 2.5 and 8.5 months, respectively (32).

#### 2.2.5 PD-L1 inhibitors

Socazolimab was evaluated in a phase I and showed ORRs similar between PD-L1 positive and negative patients (16.7% and 17.9%, respectively) (33).

#### 2.2.6 Bispecific antibody

Cadonilimab (a PD-1/CTLA-4 bi-specific antibody) and Bintrafusp alfa (an innovative bifunctional fusion protein consisting of the extracellular domain of transforming growth factor-β receptor II (TGF-βRII) fused to human IgG1 mAb of PD-L1) were evaluated in phase II studies and showed ORRs of 33% and 31%, respectively (34, 35).

The unsuccessful results of ICI monotherapies have led to studies evaluating such as ICI combination therapy, ICI and targeted therapy, antibody-drug conjugate (ADC), adoptive cell therapy, and therapeutic vaccines.

#### 2.2.7 Combination of ICI and anti-VEGF

Treatment with PD-1 mAb (Sintilimab/Camrelizumab) in combination with or without antiangiogenic drugs (Apatinib) was evaluated in 102 patients with recurrent or metastatic CC in a retrospective study and showed an ORR of 51.0%, a DCR of 66.7%, and a median PFS of 11.0 months (36).

Atezolizumab and cadonilimab (AK104) in combination with bevacizumab were tested in 2 phase II studies and showed ORRS of 0%, 73.3% (11/15), 68.8% (11/16), and 92.3% (12/13) for atezolizumab + bevacizumab, AK104 15 mg/kg, AK104 10 mg/kg and AK104 10 mg/kg + bevacizumab 15 mg/kg, respectively (37, 38).

Camrelizumab (anti PD-1 antibody) + apatinib (anti VEGFR2 antibody), Sintilimab (anti-PD-1) + anlotinib (a small molecule VEGFR-2 selective inhibitor), nivolumab + lucitanib [a tyrosinase inhibitor with multiple targets (VEGFR1-3, FGFR1-3, and PDGFRa/b)] and serplulimab (anti-PD-1) + albumin-bound paclitaxel showed in phase II studies, ORRs of 55.6% (95% CI, 40.0% to 70.4%), 59.0%, 23.5% and 57.1% and a median PFS 8.8 months (95% CI 5.6 months not reached), 9.4 months, not reported and 5.7 months, respectively (39–42).

The double-blind, randomized, phase III KEYNOTE-826, in which 617 patients received carboplatin + paclitaxel with or without bevacizumab randomized between pembrolizumab and placebo, showed improved PFS [12.4 months vs 8.2 months, HR =0.65 (95% CI: 0.53-0.79)] as well as OS (24.4 months vs 16.3 months; HR= (0.67 (95% CI: 0.54 -0.84)). The subgroup analysis does not find any benefit in patients with a CPS score <1 and those who are metastatic from the outset (43).

#### 2.2.8 ICI combination therapy

Balstilimab + zalifrelimab (anti-CTLA-4), pembrolizumab + tisotumab (ADC that targets tissue factors and releases a microtubule-disrupting agent, monomethyl auristatin E) and pembrolizumab + GX-188E (Tirvalimogene teraplasmid is an HPV-16 and HPV18 E6 and E7 therapeutic DNA vaccine) showed in phase II studies ORRs of 25.6%, 41% and 33.3% and median PFS of not reported, 5.3 months, not reported (44–46).

Nivolumab + tumor-infiltrating lymphocytes (TILs) showed for patients with low microsatellite instability (MSI) expression, PD-L1 negative metastatic CC an ORR of 25%, a median PFS of 6.1 months, and a median OS of 11.3 months (47).

### 2.3 Synthetic

Among patients with recurrent or metastatic CC, two large randomized controlled phase III trials have shown improved outcomes. The GOG-3016 trial reported improved overall survival in patients treated with ICI compared with CT alone. The KEYNOTE-826 study showed an almost one-year improvement in overall survival when combining an ICI with CT, with or without anti-angiogenic therapy, compared with CT alone. However, some studies have failed to achieve such encouraging results, probably due to the suppression of the tumour microenvironment (32, 47). RT, and especially the high-dose fractionated RT, may induce a strong antitumor immune effect. Thus, the combination of RT and ICI could lead to an improvement of the efficacy of ICI.

## 3 Abscopal effect

### 3.1 Rational of optimizing local efficiency towards remote efficiency

Since the first use of RT to treat in the early 1900s, local efficacy with local toxicity has been observed. However, in some cases, lesions located at a distance from the irradiated lesion have shown stability and even a response (48). This phenomenon was called the abscopal effect (“ab” meaning position away from, and “scopus” meaning “target”), i.e. at a distance from the target. At that time, this explanation for this effect remained unresolved. Today, we have preclinical and clinical arguments to affirm that there are interactions between irradiation and the immune system and we can exploit them.

Immunity has been identified to be a factor in response to RT. In a mouse model, tumors were implanted subcutaneously in immunodeficient (nude) and immunocompetent mice and then irradiated. Measurement of the tumor growth curves showed that the response to irradiation is totally different between an immunodeficient and an immunocompetent mouse. Indeed, the immunodeficient mouse showed no response to irradiation while the immunocompetent mouse responded. Thus, immunity could have an impact on the response to irradiation (49).

The immune mechanism is explained by the fact that a dead tumor cell will release a cascade of signals and ligands into the microenvironment and express receptors on its surface that will activate immunity. Indeed, we will observe an increase in the expression rate of the major histocompatibility complex after irradiation. Then, this process results in the release of cytokines, cell death factors and cell damage factors which increase immunogenicity and lead to interactions with dendritic cells and T cells (50).

When a tumor is irradiated, there are 2 main mechanisms. The first is direct cell death linked to apoptosis, mitotic catastrophe (particularly in the case of a p53 mutation and for tumor cells), and other types of cell death including oncosis. The second is indirect cell death linked to the activation of T lymphocytes that will destroy the surviving tumor cells (51). Thus, tumor cells that survive irradiation can be eliminated by immune cells.

Increased immunogenicity may be enabled by anti CTLA-4 and anti PD-1, PD-L1 monoclonal antibodies (52). In combination with RT, a synergy has been pre-clinically demonstrated in implanted breast cancer models in mice with an increase in local efficacy but also an effect on the non-irradiated tumor (53). Thus, the combination of RT with an immunomodulator can induce an action on non-irradiated sites at a distance, this is the abscopal effect. In fact, a tumor irradiated with a high dose will release antigens in the microenvironment which will be taken up by dendritic cells and which will migrate into the lymph nodes and lead to an effector immune response with T and B lymphocytes, which will proliferate in the lymph nodes and will destroy the tumor cells which would have survived the irradiation locally or those which are at a distance (50).

For the moment the abscopal effect is very uncommon because the tumors are well developed with immune escape mechanisms. Thus, it is difficult for RT alone to create an effective immune response, hence the interest in combining RT with ICI (54). The current goal is to generalize this rare phenomenon given its potential impact on efficacy, particularly on overall survival and progression-free survival (55).

In CC, there are very few results on the combination of ICI and radiotherapy.

### 3.2 Preclinical clinical studies

One preclinical study evaluated anti-CD40 therapy to boost the abscopal effect (56). CD40 is a costimulatory protein that binds to CD154 (CD40L) on T helper cells allowing activation of APCs and a cascade of immune effects. Studies have shown the absence of CD40 overexpression in normal cervical epithelium, whereas it was overexpressed in human papillomavirus infection and in SCC of the cervix (57, 58). Ligation of CD40 results in inhibition of tumor growth through strong apoptotic signals to cancer cells. This could be a candidate therapeutic target that would result in the production of antigens to enhance the abscopal effect (58).

In this study, TC-1 cells were injected subcutaneously into both dorsolateral flanks of each mouse to mimic metastatic cervical cancer models. Tumor growth was monitored to a treatable size of 3-4 mm in diameter. Two main results were highlighted in this study. The first one concerned the optimal irradiation dose in combination with a single dose of anti-CD40 to obtain an abscopal effect, and only the lowest dose studied, 6 Gy, showed a significant abscopal response compared to higher doses of 10 and 15 Gy. The second result concerns the number of anti-CD40 injections, the greatest abscopal effect was in the group receiving a single dose of anti-CD-40 and not in the group receiving 3 doses.

A study assessed intratumoral changes induced by radioimmunotherapy by performing single‐cell RNA‐sequencing on a pair of cervical squamous cell carcinoma samples before and during radioimmunotherapy (59). They showed that radioimmunotherapy induced changes in the tumor and immune microenvironment including transformation of epithelial cell subclusters from a malignant to a normal phenotype with some residual malignant cells, an increase in apolipoprotein E^+^ macrophages with high levels of M2 features, and a decrease in inhibition scores of residual exhausted CD8^+^ T and regulatory T cells.

### 3.3 Clinical results

A case report about a patient with recurrent cervical cancer metastatic to the liver demonstrated the benefits of combining radioembolization with pembrolizumab, the patient having achieved a complete response at 8 months. Like SBRT, radioembolization enables very high doses to be delivered in a short space of time (60).

A phase 1 study evaluated the association of RT with granulocyte-macrophage colony-stimulating factor and found in some patients treated for metastatic solid tumors objective abscopal responses but only one patient had a CC and no abscopal response was observed (61).

A phase 1 study assessed the safety of combination of cemiplimab and RT (3x9 Gy) in metastatic or recurrent CC patients who were resistant to or intolerant of platinum and taxane CT (62). The results showed that adding RT to cemiplimab did not increase grade ≥3 AE but did not improve the response rate versus single-agent cemiplimab (one PR in ten patients [10%] in both arms).

A multi-center open-label, non-randomized phase 2 study (PRIMMO), in patients with metastatic or recurrent CC, endometrial carcinoma, or uterine sarcoma, evaluated the combination of PD-1 blockade (pembrolizumab), radiation (3x8 Gy in 48 h-intervals) and repurposed compounds (i.e. drugs approved for another indication) with (immune) modulating properties (vitamin D, aspirin, lansoprazole, a proton-pump inhibitor, cyclophosphamide and curcumin) aimed at targeting TME and promoting anti-tumor immunity (63). This study found an ORR of 11.1% (depending on the criteria) of patients in at least 2nd line treatment. The primary objective of this study was an ORR with the lower bound of the 90% CI of > 10% in either cohort. Unfortunately, it did not meet its primary objective with results even lower than those found in studies testing other associations with ICIs and the rate of grade ≥3 toxicity was 55.6% (10 patients).

A phase 1 study (GOG-9929) included 21 patients with node-positive LACC to evaluate the safety of ipilimumab administration after CT. The authors reported a safety grade ≥ 3 rate of 10% (64). Moreover, the authors found that CRT alone resulted in an increase of the activation markers ICOS and PD-1 on T-cell subsets. The combination of ipilimumab and CRT led to a significant increase of both central and effector memory T-cell populations.

Unfortunely, the CALLA trial which evaluated the addition of durvalumab to standard CRT is negative on its main endpoint which is progression-free survival (HR 0.84, 95%CI 0.65-1.08, p=0.174), which also raises the question of how to optimize the association of RT with IT in this type of tumor.

## 4 Ongoing studies

As mentioned above, few clinical results are available at this time. However, based on the potential benefit of combining RT and ICIs, trials of concomitant RT with IT are currently underway for LACC but also for metastatic or recurrent CC (**Tables 1**, **2**).

**Table 1:** ongoing studies for locally advanced cervical cancer.

Trial	Phase	Treatment regimens	Primary end-point
NCT04580771	II	CRT + vaccine PDS0101 SC on days -10, 7, 28, 49, and 170	Safety and toxicity
NCT05504642	II	Induction IT (Nivolumab 3 mg/kg and Ipilimumab 1 mg/kg IV two weeks before CRT) followed by concurrent CRT and IT (Nivolumab 3mg/kg week 1, 3, 5, 7 and Ipilimumab 1mg/kg in week 5) followed by IT maintenance (Nivolumab 3mg/kg every two weeks x12 and Ipilimumab every six weeks x4)	PFS
NCT05173272	III	Neoadjuvant chemotherapy (Cisplatin 50 mg/m^2 d1 q21+ Paclitaxel 175 mg/m^2 d1 q21) combined with serplulimab (300mg d1 q21) followed by CRT and BT vs CRT + BT	PFS
NCT02635360	II	CRT + BT followed by IT (200 mg of pembrolizumab every 21 days for 3 months) or CRT + BT with concurrent IT (200 mg of pembrolizumab every 21 days for 3 months)	Change in immunologic markers following combination of study drug with CRT and Incidence of dose limiting toxicities
NCT03833479	II	CRT + BT vs CRT + BT followed by maintenance IT (Fixed 500 mg TSR-042 dose Q3W for the first 4 doses followed by a fixed 1000 mg TSR-042 dose Q6W for up to 24 months)	PFS
NCT05492123	II	CRT + BT vs induction nivolumab (1mg/kg every 3 weeks for 4 cycles)-ipilimumab (3mg/kg every 3 weeks for 4 cycles), followed by nivolumab (240mg every 2 weeks) with CRT and BT	3-year PFS
NCT03612791	II	CRT + BT vs atezolizumab (1200 mg Q3W, starting one week before EBRT (Week -1) and continued as an adjuvant for a total maximum of 20 cycles) with CRT and BT	PFS
NCT05311566	II	CRT + BT vs Camrelizumab (200mg, every 2 weeks and continued as an adjuvant) with CRT and BT	3-year OS rate
NCT04221945	III	CRT + BT vs pembrolizumab (200 mg on Day 1 of each 3-week cycle for 5 cycles during CRT followed by pembrolizumab 400 mg IV on Day 1 of each 6-week cycle (Q6W) for an additional 15 cycles.) with CRT and BT	PFS OS

CRT, chemoradiation therapy; BT, brachytherapy; IT, immunotherapy; PFS, progression-free survival; OS, overall survival.

**Table 2:** ongoing studies for recurrent or metastatic cervical cancer.

Trial	Phase	Treatment regimens	Primary end-point
NCT03614949	II	SBRT with 24 Gy in 3 fractions to participants with ≥ 2 metastatic sites and Atezolizumab 1200 mg intravenously every 3 weeks	ORR
NCT03277482	I	Durvalumab (every 4 weeks for a maximum of 13 doses over 52 weeks) + Tremelimumab (every 4 weeks for a maximum of 4 doses over 16 weeks) + RT	Maximum Tolerated Dose of RT with durvalumab and tremelimumab
NCT05310383	II	Tislelizumab (200 mg intravenously (IV) on Day 1 of each 3-week cycle (Q3W) for up to 35 cycles) plus RT in addition to CT	ORR
NCT04974827	II	Camrelizumab (200mg/3weeks) Combined with concurrent CRT in patients with cervical cancer who had recurrence of the pelvic wall after surgery ± abdominal aortic lymph node metastasis	Complete remission rate
NCT03589339	I	Intratumoral in multiple primary tumors injection of NBTXR3 followed by SABR followed by monotherapy with nivolumab or pembrolizumab	Determination of the Recommended Dose

SBRT, stereotactic body radiation therapy; ORR, overall response rate; RT, radiation therapy; CT, chemotherapy.

## 5 Perspectives

In CC, the responder rate to IT monotherapy is lower than in melanoma or in lung cancers for example. Furthermore, no objective abscopal effect was found in advanced or recurrent CC when combining RT and ICIs. Prospective randomized trials evaluating the combination of RT and ICIs are needed. However, it is also essential that studies be conducted to improve the therapeutic index of this promising association.

### 5.1 Improvement radiation therapy in combination with immunotherapy

RT alone allows a kind of reprogramming of the tumor microenvironment with the increase in pro immunogenic actors, in particular pro immunogenic myeloid populations, effector CD8 Ts, but on the other hand it also increases regulatory Ts (65, 66). Thus the current objective is to tilt the immune balance by reducing the negative effects of RT or by increasing its positive effects (67). Today, it is not a question of using IT to increase the response to RT but rather the opposite, to use this local treatment that is RT and to optimize it in order to combine it with IT and potentiate its effects.

The parameters that can be optimized in RT are i) the irradiation fields, in particular the lymph node areas, ii) the total dose and the dose per fraction, iii) the identification of new organs at risk such as the blood, iv) the therapeutic sequence with ICI v) and the irradiation of several sites simultaneously.

i) Irradiation fields and lymph node sparing

In a preclinical study using immunocompetent mouse model with tongue cancer treated by an inhibitor of CTLA-4, the authors compared efficacy between a local treatment of lymph nodes to no local treatment (68). They found that the anti-CTLA-4 allowed a tumor control which is abrogated in case of local treatment of the lymph node areas by surgery or RT. Thus, by irradiating the drainage lymph nodes of the tumor at 18 Gy, the effect of anti-CTLA-4 was abolished. Furthermore, there were significant differences in the tumor-infiltrating immune population depending on whether or not lymph node treatment was performed after anti CTLA-4 therapy. In the group without local lymph node treatment, there was a large infiltrate of CD4 and CD8 cells and few immunosuppressive myeloid cells, whereas the opposite was in case of local lymph node treatment.

In a phase II trial (69), early-stage non-small cell lung cancers (NSCLC) evaluated a neoadjuvant treatment combining durvalumab and SBRT (3x8 Gy) of the primary tumor only without including draining nodes versus durvalumab one before surgery. The combination was safe and was associated with an increase of histopathological response (53.3% versus 6.7% (p<0.001)). Interestingly, on the off-screen response, 13 patients had pre-treatment positive lymph nodes detected on PET/CT.

In the JAVELIN trial (70), there is irradiation of the primitive site and of the lymph node drainage areas at high dose (70 Gy in 35 fractions) with Avelumab. This study did not meet the primary objective (PFS) and one of the explanatory hypotheses for this failure is the lack of modification of the irradiation field to spare the lymph node drainage areas.

Thus, the question arises in the context of association with IT to exclude the first lymph node relay from RT fields.

**Figure 1:**
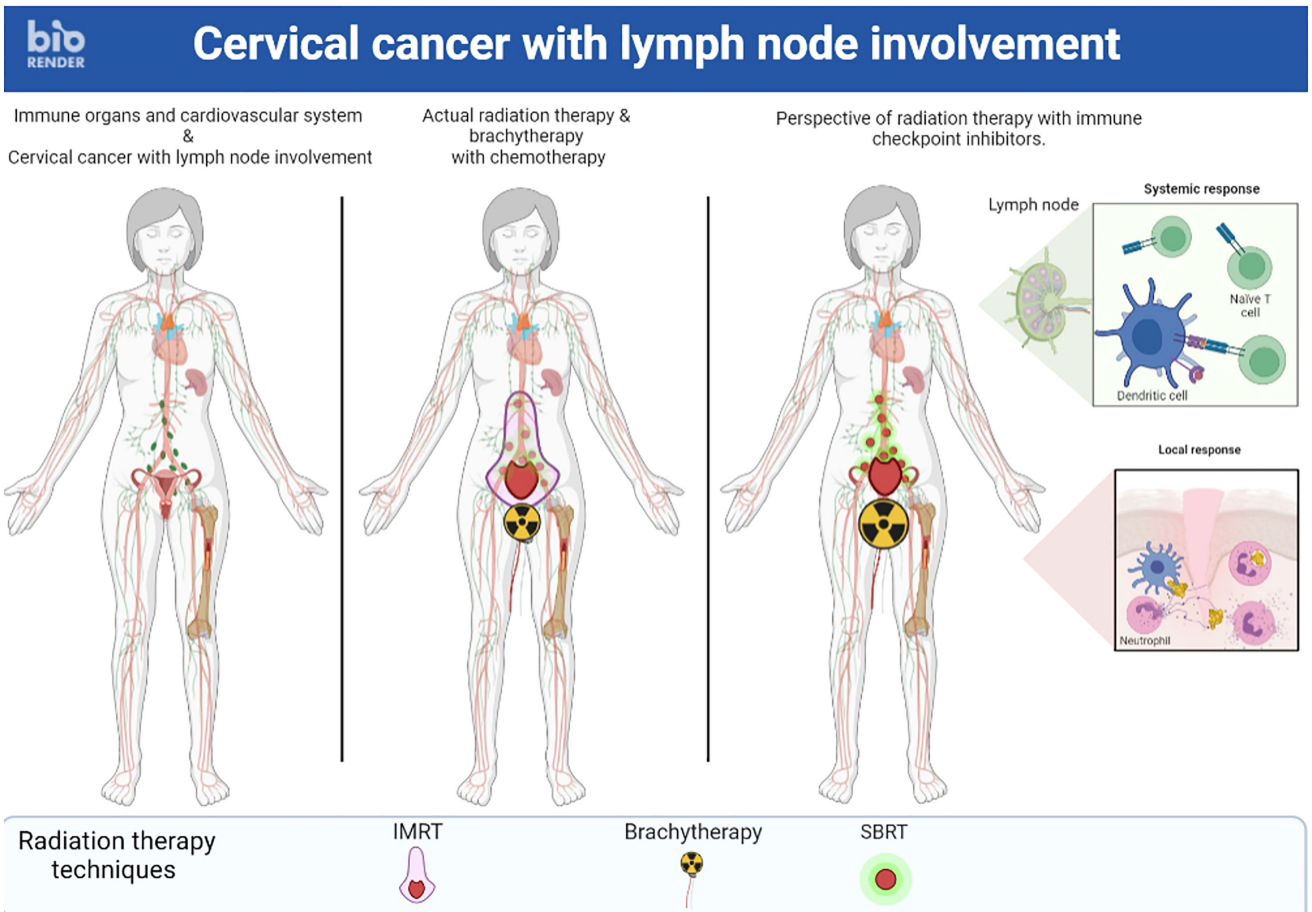
A locally advanced cervical cancer where stereotactic body radiation therapy alone on the pathological nodes combined with radiation therapy on the primary tumor with chemotherapy and immunotherapy could be an alternative.

In LACC, a randomized phase II trial evaluated the value of adding induction CT before conventional CCRT (71). Results showed a decrease in 3-year PFS (40.9% vs. 60.4%) and 3-year OS (60.7% vs. 86.8%) in the induction CT group compared with the immediate CCRT group. Two hypotheses were suggested by the authors to explain these disappointing results. First, there may be a risk of local disease progression during induction CT because of the absence of local treatment during this phase. Second, the administration of induction CT could lead to side effects that would limit the administration of CT concomitant with RT. SBRT alone on the pathological nodes combined with RT on the primary tumor with CT and IT could be an alternative (**Figure 1**).

ii) Total dose, dose by fractions and heavy particles

An important role of RT dose is to increase recruitment of the innate immune system and initiate gene transcription of the interferon that will be released in the TME, which is key to activating the immune response. It will enable the recruitment of immune cells into the TME and of dendritic cells for cross-presentation (72, 73).

*In vitro* and *in vivo* models have shown that there is more interferon response when using doses of 8 Gy than when using higher doses of 20 Gy, for example. Thus, it would seem that 3x8 Gy is the optimal scheme (74). Recently, a classification of RT regimens has been proposed according to their potential to enhance immune responses (75).

A preclinical study investigated the combination of brachytherapy (3x8 Gy) with IT (anti-PD1, anti-CD137, and/or their respective isotype controls) in mice with subcutaneous colorectal cancer at 2 sites. To evaluate a possible abscopal effect, only one tumor was irradiated. They demonstrated a response on the non-irradiated tumor, and thus an abscopal effect, only in mice receiving the BT and ICI combination (76). The clinical application of this approach could potentially provide a treatment alternative for patients being treated for CC where brachytherapy is a routinely used treatment.

One study reported the results of a combination of high-dose and low-dose RT versus high-dose RT alone in patients with IT-resistant metastatic cancer (77). This combination appeared to improve lesion-specific response in patients with immune-resistant solid tumors by promoting the infiltration of effector immune cells into the TME.

Heavy particles, such as carbon ions and protons, seem to have immune effects that could be of interest in association with ICIs (78). A multicenter Phase II trial to assess the feasibility and clinical activity of adding carbon ion radiotherapy to immune checkpoint inhibitors in cancer patients who have achieved stable disease with pembrolizumab administered as standard therapy has started (78).

iii) Identification of new organs at risk such as blood

Studies have shown that RT could induce lymphopenia resulting in poorer overall survival for many types of cancer (79). The mechanism of this lymphopenia can be direct in case of a large percentage of irradiated circulating cells making the treatment less effective. For example, in the case of an 8 cm lung cancer treated with 60 Gy in 30 fractions, a 2 Gy fraction gives 0.5 Gy to 5% of the circulating cells and 3 fractions give more than 0.5 Gy to 99% of the circulating cells (80). This suggests the value of hypofractionation and reduction of radiation fields and low doses. These results are in agreement with the recommendations that have been proposed recently (81).

The mechanisms of CRT-induced lymphopenia are being explored since it could also be caused by treatment-induced circulating immunosuppressive myeloid cells (82).

Moreover, the response of a tumor to irradiation has been shown to be impacted by neutrophilia. So hyperleukocytosis is a pejorative factor in the response to irradiation and we see a clear, linear response between the severity of the different cancers and the proportion of patients who have baseline hyperleukocytosis, which suggests the link between neutrophil count and response to irradiation (83). In the case of CC, intense spinal fixation could be seen on PET scan. Having bone marrow hyperactivation in non-metastatic cervical cancer patients is highly significantly correlated with the probability of survival. This hyperactivation is related to hyperleukocytosis and correlates with poor response, but also with tumor infiltration by myeloid-suppressive cells. This suggests a deleterious role of the myeloid compartment in the response to irradiation (84).

iv) Therapeutic sequence

It is a challenge to find the optimal therapeutic sequence in which RT and IT are administered. Anti-PD-1 administration before irradiation sensitizes T cells or more γH2ax breaks and later apoptosis are observed (85). In mice, this results in improved survival and preferential induction of the abscopal effect if IT is given after RT.

**Figure 2:**
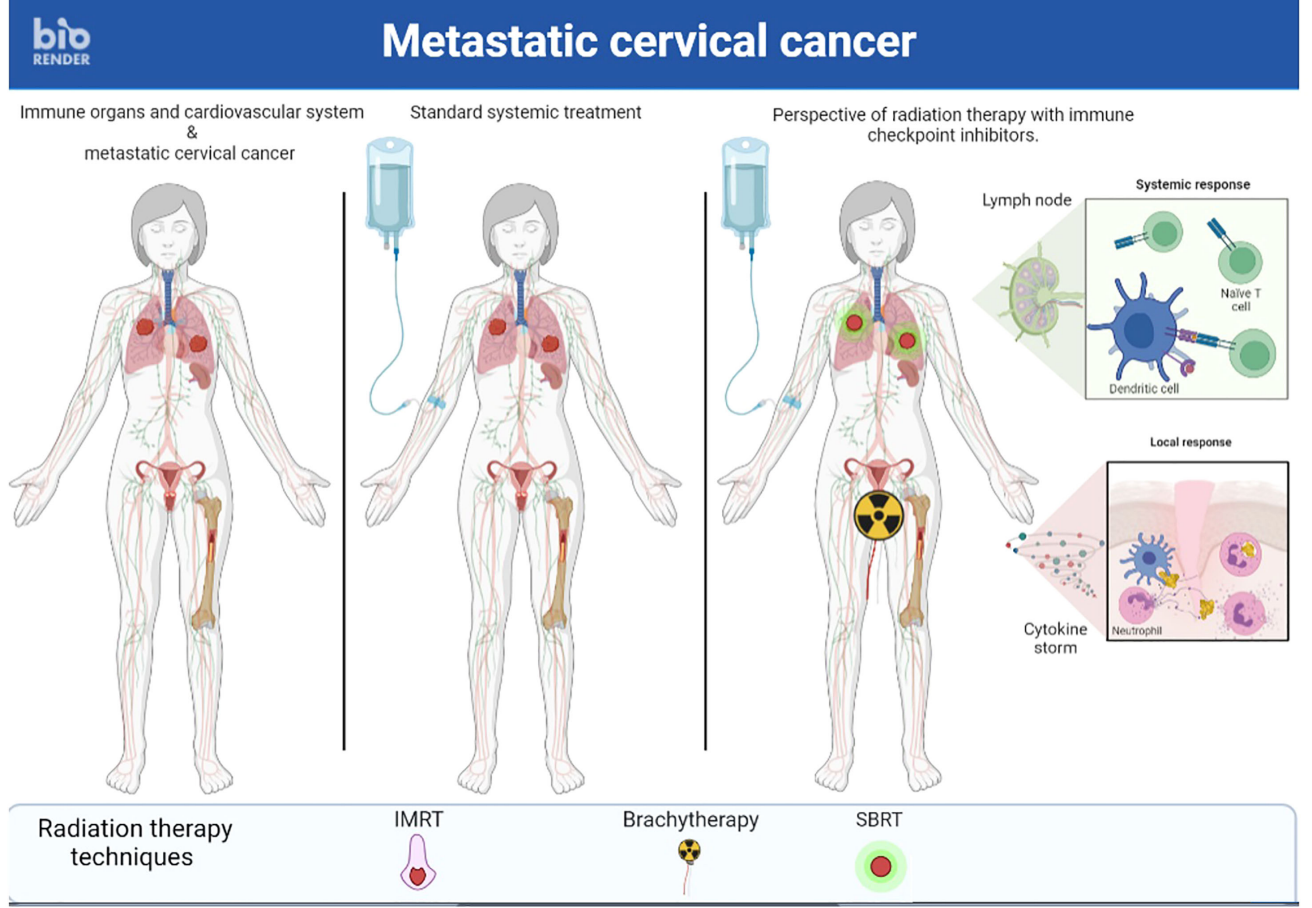
A case of metastastic cervical cancer treated with stereotactic body radiation Brachytherapy therapy multiple metastastic sites and radiation therapy followed by brachytherapy on primary tumor and immunotherapy simultaneously to enhance immune response.

**Figure 3:**
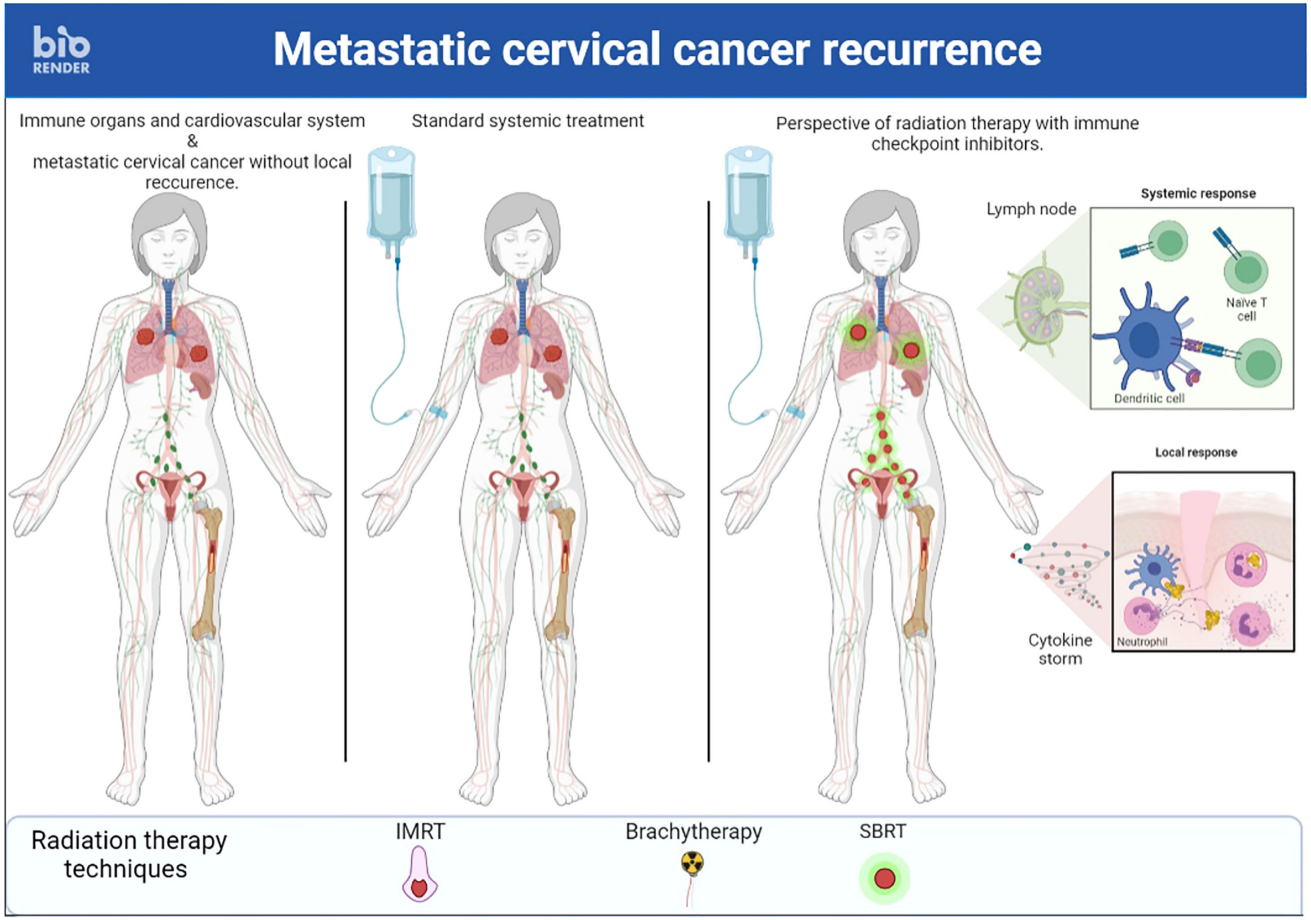
A case of recurrent cervical cancer treated with stereotactic body radiation therapy on multiple metastastic sites and immunotherapy simultaneously to enhance immune response.

v) A final approach to promote the abscopal effect would be to abandon the idea of irradiating a single metastatic site but rather to irradiate several sites simultaneously (86) (**Figures 2**, **3**). First, this RT targeting different tumor lesions could avoid T cell exhaustion by decreasing the antigenic load (87). Moreover, ICI seems to be more effective with lower volume disease (88). This multi-site RT could lead to a larger and more diversified release of tumor antigens. Finally, this RT is now feasible in the light of the many technological advances that make it increasingly accurate (89). Unfortunately, this RT is not widely available in countries where cervical cancer is most common.

### 5.2 Management of the tumor microenvironment to enhance the immunogenic side of radiation therapy

Another avenue of research to improve the therapeutic index of RT and ICI combinations would be to manipulate the TME to improve the immunogenicity of RT and reduce its immunosuppressive effect.

Potential TME targets have already been identified, such as tumor-associated macrophage (combination with granulocyte colony-stimulating factor or with a colony-stimulating factor 1 receptor antagonist monoclonal antibody), 4-1BB, a transmembrane glycoprotein presents on activated effector T cells (combination with a 4-1BB agonist monoclonal antibodies) and TGF-β (combination with a monoclonal antibody targeting TGF-β or with a bispecific antibodies targeting both PD-1 and TGF-β) (90).

PRIMMO study tried to modulate the TME in an inexpensive manner with repurposed compounds (i.e. drugs approved for another indication) with (immune) modulating properties (63). However, this study failed to improve ORR. Thus, despite the promising preliminary evidence and biological potential of these compounds, these results emphasize that additional studies are needed to obtain clinical evidence of their benefit.

The result of a preclinical study showed that cisplatin CT can enhance the abscopal effects induced by RT in association with ICI (91). This study highlights that efficiently attracting induced T cells to unirradiated tumors is an interesting alternative to obtain an abscopal effect in addition to the induction of tumor-specific T cells. This finding is particularly interesting given that cisplatin is the most widely used CT for CC.

Pre-clinical results have shown that modulated electro-hyperthermia (mEHT) associated with IT would be able to induce an immune mediated response (92). A phase III randomized controlled trial has evaluated the combination of mEHT with CRT in LACC (93). The rate of complete metabolic response (CMR) was higher in the mEHT group than in the control group 24.1% vs. 5.6% (p = 0.013), both within and outside the radiation fields. Thus, obtaining a CMR from an unirradiated tumor shows that combining mEHT with ICI and RT treatment would increase the abscopal effect. It can be assumed that RT and mEHT have a synergistic action that could be used in the treatment of metastatic disease treated with ICIs.

### 5.3 Biomarkers

To improve the ratio of ICI + RT, it is essential to identify the subpopulation of patients who benefit the most. Various grading systems and thresholds have been identified to assess PD-L1 expression by immunohistochemistry. However, these biomarkers have only been used as biomarkers in clinical trials of ICIs in CC and not in the RT + ICI combination. They include the ratio of PD-L1-stained tumor cells (TCs) to the total number of viable TCs, the combined positive score (CPS) which is defined as the total number of PD-L1-stained cells (including TCs, lymphocytes, and macrophages) divided by the number of all viable TCs, then multiplied by 100 (94).

Moreover, studies have shown that it is possible to identify patients at risk of polymetastatic progression from patients who will have oligometastatic progression using molecular biomarkers (95). Thus, one could avoid proposing RT to patients at risk of polymetastatic progression. In agreement with this concept, a study of colorectal cancer liver metastases suggested the use of a molecular classification to identify patients at risk of progression after metastasectomy (96). This could be a relevant tool to select patients with oligometastatic tumors eligible for curative treatment, i.e. who could benefit from the combination of RT and ICIs.

Finally, an ongoing study (NCT04574635) collects blood samples to assess if the DNA of HPV that causes cervical cancer can be detected in patients with CC that is new (primary), recurrent, or metastatic and are undergoing treatment with surgery, RT, CT, and/or ICI. The main objective is to predict response of the cervical cancer to treatment and detect recurrent cancer sooner.

## 6 Conclusion

Currently, there is no clinical proof of abscopal effect in advanced, recurrent or metastatic CC with the combination of RT and ICI. Thus, the results of the ongoing prospective randomized trials combining RT and ICIs will be very informative. Research avenues concern new strategies to improve the therapeutic index of this combination including manipulation of the tumor microenvironment, by combining new drugs such as 4-1BB agonist monoclonal antibodies or bispecific antibodies targeting both PD-1 and TGF-β, and reflection on the type of RT to be combined with ICIs, mainly techniques enabling high doses to be delivered per fraction such as SBRT or brachytherapy.

## Author contributions

Conceptualization, FL and LO; methodology, FL and LO; investigation, FL and LO; data curation, FL, CM, ER, ED and LO; writing—original draft preparation, LO and FL; writing—review and editing, FL, CM, ER, ED and LO. All authors have read and agreed to the published version of the manuscript.
